# Tdrd3-null mice show post-transcriptional and behavioral impairments associated with neurogenesis and synaptic plasticity

**DOI:** 10.21203/rs.3.rs-2597043/v1

**Published:** 2023-03-02

**Authors:** XingLiang Zhu, Yuyoung Joo, Simone Bossi, Ross McDevitt, Aoji Xie, Yue Wang, Yutong Xue, Shuaikun Su, Seung Kyu Lee, Nirnath Sah, Shiliang Zhang, Rong Ye, Alejandro Pinto, Yongqing Zhang, Kimi Araki, Masatake Araki, Marisela Morales, Mark Mattson, Henriette van Praag, Weidong Wang

**Affiliations:** National Institutes of Health; National Institute on Aging; National Institute on Aging; National Institute on Aging; National Institute on Aging; National Institute on Aging; National Institute on Aging; National Institute on Aging; National Institute on Aging; National Institute on Aging; Florida Atlantic University; Florida Atlantic University; National Institute on Aging; National Institute on Aging; Institute of Resource Development and Analysis, Kumamoto University; Institute of Resource Development and Analysis, Kumamoto University; National Institute on Drug Abuse; Department of Neuroscience, Johns Hopkins University School of Medicine; Florida Atlantic University; National Institute on Aging

**Keywords:** Top3b, Tdrd3, neurological disorders, neurogenesis, myelination

## Abstract

The Topoisomerase 3B (Top3b) - Tudor domain containing 3 (Tdrd3) protein complex is the only dual-activity topoisomerase complex in animals that can alter the topology of both DNA and RNA. *TOP3B* mutations in humans are associated with schizophrenia, autism and cognitive disorders; and *Top3b*-null mice exhibit several phenotypes observed in animal models of psychiatric and cognitive disorders, including impairments in cognitive and emotional behaviors, aberrant neurogenesis and synaptic plasticity, and transcriptional defects. Similarly, human *TDRD3* genomic variants have been associated with schizophrenia, verbal shorten-memory and learning, and educational attainment. However, the importance of *Tdrd3* in normal brain function has not been examined in animal models. Here we built a *Tdrd3*-null mouse strain and demonstrate that these mice display both shared and unique defects when compared to *Top3b*-null mice. Shared defects were observed in cognitive behaviors, synaptic plasticity, adult neurogenesis, newborn neuron morphology, and neuronal activity-dependent transcription; whereas defects unique to *Tdrd3*-deficient mice include hyperactivity, changes in anxiety-like behaviors, increased new neuron complexity, and reduced myelination. Interestingly, multiple genes critical for neurodevelopment and cognitive function exhibit reduced levels in mature but not nascent transcripts. We infer that the entire Top3b-Tdrd3 complex is essential for normal brain function, and that defective post-transcriptional regulation could contribute to cognitive impairment and psychiatric disorders.

## Introduction

Topoisomerases resolve topological problems generated during DNA replication, transcription, and chromosome segregation. Top3b has the unique capacity to resolve topological problems for both DNA and RNA. Increasing evidence shows that Top3b works with Tdrd3 in a conserved Top3b- Tdrd3 dual-activity topoisomerase complex in animals^1–5^. Human genetic studies have shown that *TOP3B* deletion or mutations are associated with psychiatric and cognitive disorders, including schizophrenia, autism, epilepsy, and intellectual disability^2, 6–10^; consistent with critical *TOP3B* function in normal brain function and neurodevelopment. This inference is supported by analyses of cultured neurons^1^ and several animal models, including mouse^11, 12^, zebra sh^13, 14^, and *Drosophila*^1, 5^. All showed that *Top3b* deficiency can lead to neuronal and/or behavioral abnormalities. Specifically, our group has observed that *Top3b*-null mice^15^ display behavioral phenotypes related to psychiatric disorders and cognitive impairment, as well as impairments in hippocampal neurogenesis and synaptic plasticity^11^.

On DNA, Top3b -Tdrd3 has been reported to promote transcription^11, 16^, R-loop resolution^17–19^, siRNA-guided heterochromatin formation and transposon silencing^20^. At the RNA level, Top3b-Tdrd3 has been shown to associate with RNA stress granules, localize with the mRNA translation machinery^1, 2, 4^, and regulate mRNA translation and turnover^21^. Tdrd3 functions in several ways in the Top3b-Tdrd3 complex. First, it can enhance the binding and topoisomerase activity of Top3b on both DNA and RNA substrates^3,22^. Second, Tdrd3 can act as a scaffold to anchor Top3b to other proteins functioning on DNA or RNA, including RNA polymerase II (pol II)^11, 16, 23^, histones^24^, DExH-box helicase 9 (DHX 9)^19^, FMRP (Fragle X mental retardation syndrome protein)^1, 2^, exon junction complex^2, 25^, poly-ribosomes^1, 2^, RNA stress granules^1, 2^, and RNA-induced silencing complex^20^. The Tudor domain of Tdrd3 is crucial in mediating these interactions by recognizing methylated arginine residues in its partner proteins. Third, Tdrd3 and Top3b are mutually stabilizing, as depletion of either reduces the level of the other^1, 11, 17, 21^. These finding suggest that the two proteins act coordinately in a molecular machine to solve topological problems of DNA or RNA. In support of this notion, we recently found that the two proteins coordinately regulate transcription^16^, translation and turnover of mRNAs^21^ in isogenic *TOP3B*-null and *TDRD3*-null human cell lines.

One interacting partner of Top3b-Tdrd3 complex, FMRP, is silenced in Fragile X Mental retardation syndrome, which is the known leading cause of autism^26^. The findings that two major interacting partners of TDRD3, TOP3B and FMRP, are linked to psychiatric and cognitive disorders imply that TDRD3 itself could be associated with the same disorders. Consistent with this hypothesis, independent genome-wide association studies (GWAS) have reported association between several single nucleotide variants (SNVs) within or near *TDRD3* genomic locus and schizophrenia^27, 33^; cognitive dysfunction (such as verbal short-term memory and learning)^28, 29^, language impairment or delay^30–32^, and education attainment^29, 33, 34^. However, to date, no *Tdrd3*-deficient animals have been reported to display neurological and behavioral abnormality. This is in contrast to Top3b and FMRP, both of which have been extensively analyzed in various animal models, showing phenotypes consistent with impaired brain function^11, 12,13, 14,26^. Only one study in *Drosophila* showed that Tdrd3 can genetically interact with FMRP to promote eye formation^1^, implying that Tdrd3 could function in neurodevelopment.

Here we establish a new *Tdrd3*-null mouse model to test for its importance in normal brain function. We found that *Tdrd3*-null mice exhibit behavioral and neurological defects that overlap but are not identical to those of *Top3b*-null mice. The shared defects include cognitive functions, synaptic plasticity, neurogenesis, and neuron morphology, indicating that the entire Top3b-Tdrd3 complex is indeed critical for normal brain function. The defects found only in *Tdrd3*-null mice include reduced anxiety and myelination as well as elevated neuron complexity, suggesting that Tdrd3 may also have function(s) independently of Top3b. Interestingly, multiple genes critical for neurodevelopment and mental functions exhibit reduced levels in mature but not nascent transcripts, suggesting that defective post-transcriptional regulation could contribute to cognitive and neurological disorders.

## Materials And Methods

### Mice

The *Tdrd3*-null mouse line was generated at Kumamoto University by targeted disruption of mouse *Tdrd3* gene on a C57BL/6J background. Adult (3–10 month old) mice were used in behavioral tests; 10-week-old mice were used for all other experiments. All animal procedures were approved by the NIA animal care and use committee (ACUC) and following the NIH animal guidelines.

### Behavioral tests

Morris water maze: This test was carried out as previously described^11^, but two probe tests were carried out at both 4 and 24 hours after training. Three such trials were excluded from analysis due to excessive floating (more than 20 seconds immobile prior to first swimming). To avoid confounding influence from remaining floating in training and probe trials, measures of path length rather than escape latency were used for analysis.

#### Spontaneous alternation.

Mice were allowed to freely explore for 15 minutes in an opaque plexiglass maze containing four arms (5 cm × 30 cm, height 120 cm) radiating from the center at 90° angles. Arm entries were automatically detected by an overhead camera and ANY-Maze software (Stoelting; Wood Dale, IL). Alternations were scored as sequences of entries to four unique arms without repeats (e.g., ABCD or ABDC, but not ABCA). Alternation rate was calculated as number of alternations divided by number of opportunities for alternation. One mouse failed to leave the arm it was initially placed in and was excluded from analysis.

#### Fear conditioning:

Procedures were modified from previous reports^11^. In brief, mice were trained with three tone-shock pairings, then tested for context at 24 hours and for auditory cue at 48 hours post-training. Freezing was detected by camera and scored automatically with Video Freeze computer software (MED-Associates; St Albans, Vt).

#### Marble burying:

Mice were placed in a 22 × 38 × 18 cm clear plastic chamber containing 5 cm of fresh bedding and 20 regularly spaced 13mm glass marbles. After 30 minutes, mice were gently removed, and the number of marbles covered more than 2/3 in bedding material were counted.

#### Additional tests:

Open field test, zero maze, light-dark box, buried food test, 3-chamber sociability, reciprocal social interaction, acoustic startle, and prepulse inhibition were carried out as previously described^11, 35^.

### Electrophysiological tests

The procedures followed a previous protocol^11^. 6–8 slices from 5 WT and 6 *Tdrd3*-null mice were used.

### BrdU labeling of adult neural stem cells

For short-time proliferating analysis, Bromodeoxyuridine (BrdU, Sigma) was diluted in PBS to make a 10 mg/ml stock solution. The 10-week-old mice were given single dose 200 mg/kg BrdU intraperitoneally (*i.p*.) and sacrificed after 2 hours^36^. For long term analysis, the mice were given 4 consecutive doses (50 mg/kg each time) by *i.p*. with a 12-hour interval and sacrificed after 4 weeks^36^. The collected brains were tested by immunofluorescent staining as bellows (IF parts). 3–4 WT and 3–4 *Tdrd3*-null mice were used for each analysis.

### Preparation of retrovirus and stereotaxic surgery.

The experiments were performed as previously described^11^. Specifically, mice received intracerebral injection of vector and were recovered. The mice were breed for another month to allow for the diving cells to become new neurons, then were sacrificed and imaged (brain slices). 7 WT, 5 *Top3b*-null, 4 *Tdrd3*-null male mice were used for retroviral injection.

### Immunofluorescent staining (IF)

Briefly, after anesthetized by isoflurane, mice were perfused by PBS and 4% paraformaldehyde sequentially. Then brains were isolated and incubated overnight at 4°C in 4% paraformaldehyde. After being immersed and incubated in 30% sucrose for 2 days at 4°C, brains were embedded in OCT (optimal cutting temperature) medium at −80°C for at least 30 mins, cryo-sectioned by 30 μm setting, and washed 3 times in PBS.

For BrdU staining, brain slide was incubated at 37°C, 20 min, then samples were circled by ImmEdge Pen for another 10 min. The slide was washed with PBS for 3 times, permeabilized by 0.2%Triton X-100 permeabilization buffer (in PBS) and incubated for 15–20 minutes at room temperature. Then the slide was treated by 1N HCl, 30 minutes on ice, followed by 2N HCl 30 minutes at 37°C, and then was neutralized by 0.1N borate sodium buffer, pH 8.5, 20 minutes at room temperature. Afterwards, the slide was washed with PBS buffer for 3 times.

Tissues were incubated at room temperature for 1 hr in blocking solution (10% normal goat serum and 0.1% Triton X- 100 in PBS). Tissues were then incubated overnight at 4°C with primary antibodies. The antibodies we used were rabbit anti-GFP conjugated with Alexa Fluor488 (1:200; Life technologies, A-21311), anti-BrdU (1:50, Abcam ab6326), anti-ki67 (1:200, Abcam, ab15580), anti-GFAP (1:250, Abcam, G3893), anti-Sox2 (1:400, Abcam, ab97959), anti-Parvalbumin (1:100, Millipore, MAB1572, anti-retrieval at pH6.0). The samples were washed 3 times with PBS and incubated at room temperature for 2 hr with a secondary antibody: Fluorescence anti-Mouse Alexa Fluor 488 (1:1000, Abcam, ab150113), anti-Rat Alexa Fluor 568 (1:1000, Invitrogen, A-11077), anti-rabbit Alexa Fluor 647 (1:1000, Abcam, ab150079) and DAPI (working concentration 300 nM, Life technologies, D1306). Then the slides were mounted by ProLong^™^ Glass Antifade Mountant (Thermo sher, P36982) and imaged by confocal microscope, ZEISS LSM 780 or 880. The cell numbers were counted in 3–4 slices from each mouse. Cell density is calculated by the cell count in DG zone on each slide/brain section thickness (30 μm).

### RNAscope

Here we used RNAscope Multiple Fluorescent Assay kit V2 from (ACDBio, REF323110) reported previously^37^. The main procedure followed instructions (DOC NO., USM-323100). Briefly, after the 24 h 4% PFA fixation and 48 h 30% sucrose dehydration, the brains were embedded into OCT and then cryo-sectioned at 8 μm. Then samples were dehydrated by 50%, 70%, 100% ethanol and treated by Hydrogen Peroxide. The target retrieval was done by mildly boiled ddH_2_O with temperature monitoring. After being treated by Protease III, the samples were hybridized by probe (Mm-Tdrd3-C2, REF1032531-C2; Positive control mixture from mouse genes, REF320881; Negative control mixture from bacteria genes, REF320871). Then the signals were amplified by triple hybridizations in HybEZ^™^ Oven. The fluorescent signals were developed by incubated with Opal^™^ 570 (Akaya Bioscience Reagent kit, Part #: OP-001003) with 1:1500 dilution. Following that, the slides were counterstained by DAPI and mounted using Prolong Gold Antifade Mountant provided by the kit.

### Western Blotting

The procedures are followed by previous protocol^38^. The primary antibodies used were Top3b (1:2000, Sigma Aldrich, WH0008940M1–100UG), Tdrd3 (1:2000, Cell Signaling Technology, REF5942S), GAPDH (1:2000, Cell Signaling Technology, REF2118s).

### TUNEL staining

The procedure followed the In Situ Cell Death Detection Kit, Fluorescein kit (Roche, 11684795910) protocol. Briefly, the sections were air-dried at room temperature overnight, then were fixed with a 4% PFA in PBS for 20 min at RT. After being washed 30 min with PBS, the samples were incubated in 0.1% Triton X-100 in 0.1% sodium citrate for permeabilization about 2 min on ice. For positive control, PBS-rinsed slides were incubated with DNase I recombinant (40 U/ml) in DNase I buffer for 10 min at RT then treated by solution 1 and solution 2 mixture. Negative control was treated the same but no terminal deoxynucleotidyl transferase (solution 2). Samples were then treated by solution 1 and solution 2 mixture. All the treated samples were incubated in a humidified box for 60 min at 37°C in the dark. Later, the slides were rinsed 3 times with PBS. Following that, rinsed slides were incubated with DAPI for 15 mins at RT. The samples were washed for 3 times with PBS and then mounted with Prolong Gold Antifade Mountant. Samples were analyzed under a fluorescence microscope (10 slices for 16.5 um in Z stack, LSM 880).

### Black Gold II Myelin staining

The myelin staining followed the instructions of kit (Biosensis, TR-100-BG). Briefly, the sectioned samples were air-dried at 37°C for 2 h on a slide warmer until thoroughly dry. Then they were washed in distilled water for 2 minutes, and incubated in preheated solution A for about ~ 12 minutes in a clean covered Coplin jar in water bath, 60–65°C. The slides were rinsed in distilled water for 2 minutes and incubated in 1 X Solution B for 3 minutes. Following that, the slides were rinsed in distilled water for three times, each 5 minutes. The samples were then dehydrated via nature air-drying and immersed in xylene for 1–2 minutes to total remove water. Finally, the samples were mounted using a non-aqueous mounting media DPX (Sigma, 06522–100ML) and imaged by a lightscope.

### RT-qPCR

Half mouse brain was homogenized and removed 100 μL for RNA extract by Trizol (Invitrogen, 15596026). The sample was centrifuged the lysate for 5 minutes at 12,000 × g at 4°C, then transferred the clear supernatant to a new tube. 0.2 mL of chloroform per 1 mL of TRIzol was used for lysis. 12,000 × g, 10 min, 4°C. After that, the upper layer was transferred to a new tube and then the solution was added 600 μL acid phenol: chloroform, mixed and incubated at RT for 5 minutes. 12,000 × g, 10 min, 4°C. The upper aqueous phase was transferred into new tube. After being added 2–3 μL GlycoBlue^™^ Coprecipitant (Catalog: AM9515), the RNA was pelleted at 18000 rpm, 15 min, 4°C. The pellet was washed by 1 mL pre-cold 75% ethanol twice, air-dried and resuspended in 20–50 μL of RNase-free water. 1 μg RNA was reverse transcribed using Taqman Reverse Transcription Reagents (Applied Biosystems, N8080234). The cDNA was diluted to 1/5 and used as a template to perform qPCR with SYBR Green PCR Master Mix (Applied Biosystems, 4309155). The PCR primer sequences were listed in supplementary tableS4. The gene expression relative to *Gapdh* was calculated by 2^−ΔΔCT^ method^39^.

### RNAseq

The sample preparation and sequencing steps followed published protocols^11, 38^.

### PROseq

The procedure followed published protocol^40^ with small modifications. Briefly, the brain tissues were prepared by permeabilization buffer (10 mM Tris-HCl, pH 7.4, 300 mM sucrose, 10 mM KCl, 5 mM MgCl2, 1 mM EGTA, 0.05% Tween-20, 0.1% NP40 substitute, 0.5 mM DTT, one tablet of protease inhibitors cocktail per 50 ml and 4 units per ml RNase inhibitor) and co-incubated with 2 × nuclear run on buffer for 10 min at 37°C, 200 rpm. RNA was extracted by Trizol LS (Invitrogen, #10296028) and fragmented by 5 μl of ice-cold 1 N NaOH for 10 min on ice. After being neutralized with 25 μl of 1 M Tris-HCl, pH 6.8, the RNA was precipitated again with isopropanol. Biotin RNA was enriched by prewashed Dynabeads^™^ M-280 Streptavidin beads (Invitrogen, #11206D), washed by high salt buffer, binding buffer, low salt buffer separately, and then extracted on beads by Trizol and ZYMO RNA clean & concentrator (Zymoresearch, #R1016). RNA was ligated with 1 μl of 100 μM VRA3 RNA adaptor at 20°C overnight. Then biotin RNA was enriched and extracted again. RNA was modified in 5’ end by RppH (NEB, #M0356S) and performed hydroxyl repair by T4 PNK (NEB, #M0201L). After being extracted by ZYMO RNA clean & concentrator (Zymoresearch, #R1016), RNA was ligated with 1 μl of 100 μM VRA5 RNA adaptor at 20°C overnight. Biotin RNA was enriched and extracted for the third time. The extracted RNA was used to perform reverse transcription, PCR amplification and then sent to sequence.

### Transmission electron microscopy (TEM)

10-week male mice were perfused with perfusion/fixation buffer (2% paraformaldehyde, 2% glutaraldehyde, 15% picric acid in 1 × PB). Brains were post-fixed with perfusion/fixation buffer for 2 hours at 4°C, followed by 2% paraformaldehyde in 1 × PB for 16 hours at 4°C. Then brains were sliced coronally using animal mold, and the sliced blocks containing corpus callosum were cut into small blocks for obtaining the coronal view of axon. Thereafter, blocks were collected in 1 × PB buffer, fixed with 0.5% osmium tetroxide and contrasted in 1% uranyl acetate. After that, samples were dehydrated through a series of graded ethanol and with propylene oxide, and embedded in Durcupan ACM epoxy resin (Electron EMS, Fort Washington, PA). Resin-embedded samples were polymerized at 60°C for 2 days. After resin-embedded, 60 nm thick sections were cut with an ultramicrotome UC7 (Leica Microsystems Inc., Buffalo Grove, IL) using a diamond knife (Diatome, Fort Washington, PA). Ultrathin sections were collected on formvar-coated single slot grids and counterstained with Reynolds lead citrate. The ultrathin sections were examined and imaged using a Transmission Electron Microscope at 80,000 V (6800X and 9300X).

### Bioinformatical analysis

RNAseq. RNAseq raw data were removed adaptor by TrimGalore and checked by FastQC. Trimmed data were mapped using RSEM with the STAR alignments^41^. The bedgraph files were produced by BEDTools. The above programs were run in Biowulf in NIH. Downstream analysis was done by R using Rstudio. The differentially expressing genes were analyzed mainly by DESeq2 package and gene annotations were performed by clusterProfiler, DOSE, ReactomePA and MeSHDbi packages (Foldchage > 1.3, p-value < 0.05).

PROseq, The adaptors were removed and checked as RNAseq. Fastq files were mapped by bowtie2. Mapped data were sorted and indexed by samtools. Bedgraph les were produced by BEDTools. Counts were calculated by featureCounts in subread^42^.

### Imaging and statistical analysis.

**Figure 1:**
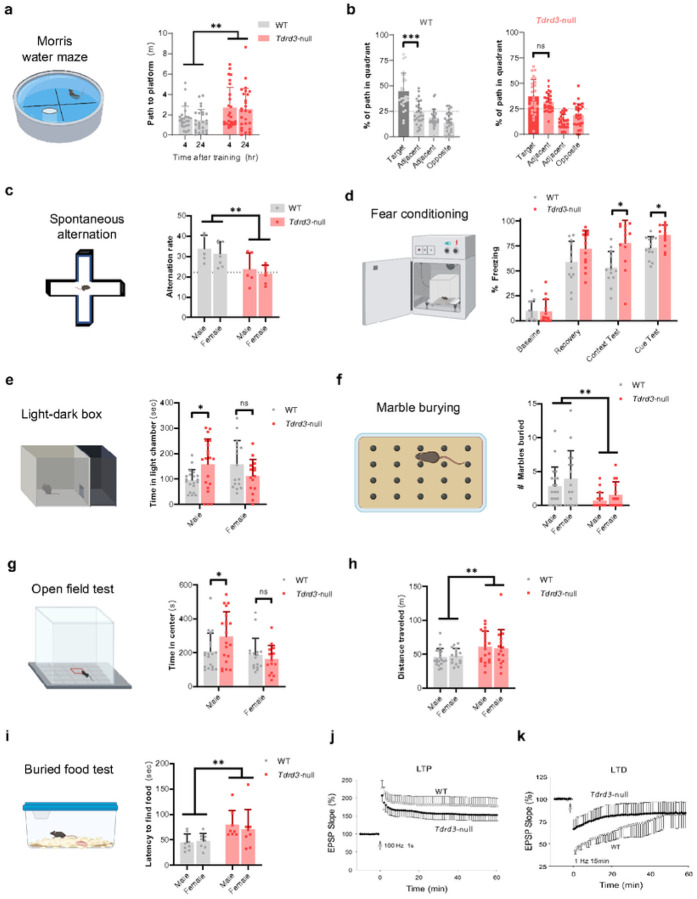
*Tdrd3*-null mice show impaired spatial memory, anxiety, olfactory sensitivity, and synaptic plasticity. **a-c** *Tdrd3*-null mice show memory impairments in the Morris water maze. a Path length until first crossing of the platform’s former location during probe tests at 4 and 24 hours post-training. ** p<0.01 main effect of genotype. **b-c** Searching preference in quadrants during 24-hour probe test for (b) wild-type and (c) *Tdrd3*-null mice. *n*=26–28 per genotype. Dashed lines indicate random chance. No sex differences were observed; for visual simplicity, water maze data is collapsed across sexes. *** p<0.001 Dunnett’s post-hoc. **d** *Tdrd3*-null mice show reduced tendency to enter least-recently visited arm during free exploration of a 4-arm maze. Dashed line indicates random chance. *n*=12/genotype. ** p<0.01 main effect of genotype. **e** Male *Tdrd3*-null mice are normal on fear training day at baseline and after 3 tone-shock pairings (“recovery”), but show increased freezing when later presented with shock-associated contextual or auditory cues. *n*=13/genotype. * p<0.05 Sidak post-hoc test. **f** Male but not female *Tdrd3*-null mice show less avoidance of the brightly lit compartment in a light-dark box. *n*=35–36/genotype. *, p<0.05 Sidak post-hoc test. **g** *Tdrd3*-null mice show a reduced anxiety-like digging response when placed in a novel environment, as assessed by fewer marbles buried. *n*=35–26/genotype. ** p<0.01 main effect of genotype. **h-i** Male but not female *Tdrd3*-null mice show less avoidance of the center of an open field in open led test (h). *Tdrd3*-null mice are more active compared to WT mice (i). n=35–36/genotype. * p<0.05 Sidak post-hoc test. ** p<0.01 main effect of genotype. **j** Tdrd3-null mice take longer to find food when guided by olfactory cues. *n*=16/genotype. ** p<0.01 main effect of genotype. **k-l** *Tdrd3*-null mice show impairments in synaptic plasticity, as being suggested by abnormal excitatory post-synaptic potentials (EPSP) from field recordings of hippocampal slices during induction of (k) long-term potentiation (LTP) or (l) long-term depression (LTD). Data presented as mean + SD (a-j), mean ± SE (k-l). *p*-values < 0.05, 0.01, 0.001 are marked as: *, **, ***; *p*-value > 0.5 is marked as no significance (ns). One-way ANOVA was performed in (b,c). Mixed effects model was performed in (a). Repeated measures ANOVA was performed in (e). 2-way ANOVA with factors sex and genotype was performed in (d,f,g,h,I,j).

The illustration of behaviors in Fig. 1 and model in Fig. S9b were created with BioRender (BioRender.com). Quantification of 50 to 60 min LTP magnitude compared with the 10 min baseline, Data: mean ± SEM. Statistical significance was performed with two-tailed Student’s t test, ANOVA, or mixed effects model (restricted maximum likelihood). Dunnet’s, Tukey’s or Sidak’s multiple comparisons tests were used following significance in ANOVA or mixed effects model tests. Several outlier data points were excluded in behavior data, using outlier criteria of > 3 standard deviations outside of group mean. *p < 0.05, **p < 0.01, ***p < 0.001.The imaged figures were analyzed by ZEN (blue version) from Zeiss and Image J (Fiji) from NIH. Statistical significances were assessed using Student’s t test using GraphPad or R, the figures were draw by Graphpad or R, too. The bedgraph files in sequencing were examined by IGV. Tracing of dendrites and spines of GFP positive adult-born neurons was performed using Bitplane Imaris software (Oxford instruments) and Image J (FIJI distribution, NIH) with SNT plugin. The traced neurons were compared and draw by Inkscape (https://inkscape.org/).

## Results

### Tdrd3 -null mice displays elevated embryonic lethality

To investigate the function of *Tdrd3* in mouse brain, we first analyzed its mRNA expression pattern by RNAscope. We found that *Tdrd3* mRNA is widely expressed throughout the brain and is especially enriched in the hippocampus (Fig. S1a). These findings are similar to those in the Allen Brain Atlas (https://mouse.brain-map.org/static/atlas), which detected enrichment of Tdrd3 mRNA in hippocampus, where Top3b is already known to function^11^.

We next developed a *Tdrd3*-null mouse line using gene-trap (GT) strategy^43^. Screening by rapid amplification of cDNA 5’-ends (5’-RACE) and long PCR of genomic DNA identified a mouse line in which the GT vector is inserted in the first intron of *Tdrd3* genomic locus (Fig. S2a-c). RT-qPCR and Western Blot analysis revealed that the levels of *Tdrd3* mRNA and protein are undetectable in the brain extracts from the homozygous mice compared to those of WT mice (Fig. S2d), confirming that the *Tdrd3* gene is inactivated. Western blotting also showed that the level of Top3b protein is concomitantly reduced to near background, consistent with previous findings that the two proteins are mutually dependent for their stability^1, 11^.

The *Tdrd3*-null mice developed to maturity at a rate similar to wildtype littermates. This resembles the development of *Top3b*^−/−^ mice^15^ as well as a Tdrd3-GT mouse line previously reported^17^. However, the observed percentage of *Tdrd3*^−/−^ newborn mice in litters from heterozygous male and female mice is about 12% (Fig. S2e), 2-fold fewer than the expected 25%. In comparison, the percentage of *Top3b*^−/−^ newborn mice was 20%, similar to the expected Mendelian ratio (< 1.5-fold difference). The phenotypic difference between *Tdrd3*^−/−^ and *Top3b*^−/−^ mice suggests that the two proteins may have independent functions, with Tdrd3 but not Top3b strongly affecting embryo viability.

### Tdrd3- null mice are impaired in learning and memory tasks

We gave *Tdrd3*-null mice a panel of behavior tests to examine whether they exhibit cognitive and anxiety abnormalities comparable to *Top3b*-null and other mouse models of psychiatric disorders. In the Morris water maze, WT and *Tdrd3*-null mice learned the location of a hidden platform at similar rates, with trends for impairment in null mice emerging only in the last two days of invisible platform training (Fig. S3a). We then conducted two probe tests at 4- and 24-hours post-training to assess memory for the platform location. In both trials, null mice showed reduced precision in their searching strategies, evidenced by traveling longer distance until the first visit to the platform location (Fig. 1a; main effect of genotype *F*_1,98_=10.5, *p* = 0.002). Wild-type mice showed a more selective bias for the target quadrant compared to other non-target quadrants (Fig. 1b, left; the distance traveled within the target quadrant is significantly longer than those in all three non-target quadrants; *F*_1.75,43.75_ =23.1, *p* < 0.0001; target vs other quadrants *p* = 0.0008, *p* < 0.0001, *p* < 0.0001), whereas *Tdrd3*-null mice searched equally in the target and one of adjacent non-target quadrants (Fig. 1b, right; *F*_1.56,40.64_=18.5, *p* < 0.0001; target vs other quadrants *p* = 0.5 (adjacent, 2nd column), *p* = 0.004, *p* < 0.0001). There were no sex differences in any of these measures (Fig. S3b), indicating that both male and female null mice have impaired spatial memory. These findings are consistent with human GWAS data that Tdrd3 is associated with cognitive function^28,29^.

We next assessed spatial working memory in a continuous spontaneous alternation task that evaluates the tendency of mice to explore their least-recently visited arm of a radial maze. *Tdrd3*-null mice had a significantly lower alternation rate (Fig. 1c; main effect of genotype *F*_1,20_=14.8, p = 0.001) and did not perform above random chance^44^ (*t*_11_ = 0.10, p = 0.92). Because both of these tasks are hippocampus dependent^45, 46^, these results are indicative of defective hippocampal function in *Tdrd3*-null mice, as has been observed in *Top3b*-null mice^11^. In support of this indication, *Tdrd3*-null mice were also hyperactive in an open field test (Fig. 1h, main effect of genotype *F*_1,67_=8.2, p = 0.006), a hallmark of animals with hippocampal lesions^47^.

In a fear conditioning task performed in males, *Tdrd3*-null mice were normal throughout a training session but showed heightened conditioned freezing during tests of context and cue at 24 and 48 hours post-training, respectively (Fig. 1d; genotype × stage interaction *F*_3,72_=3.5, *p* = 0.02; context test *p* = 0.02, cue test *p* = 0.03). This pattern of results is again very consistent with that seen in *Top3b*-null mice^11^, indicating that the Top3b-Tdrd3 complex is required for normal associative fear learning.

### Tdrd3- null mice exhibit reduced anxiety-like behaviors

Top3b-null mice display heightened anxiety-like behavior in several behavior tests^11^, a phenotype prevalent in patients and animal models of schizophrenia and autism^48, 49^. In the light-dark box, avoidance of a brightly-lit compartment was affected by *Tdrd3*-null in a sex-dependent manner, with reduced avoidance seen only in males (Fig. 1e; genotype × sex interaction *F*_1,67_=8.3, *p* = 0.005; male wt vs *Tdrd3*-null *p* = 0.03, female wt vs *Tdrd3*-null *p* = 0.2). Transitions between the chambers showed similar trends but did not reach statistical significance (Fig. S3c). In an open field test, a similar pattern of results was seen in avoidance of the center (Fig. 1g; genotype x sex interaction *F*_1,66_=4.2, *p* = 0.04; male wt vs *Tdrd3*-null *p* = 0.04, female wt vs *Tdrd3*-null p = 0.8). Although increased time in the center of open field could reflect reduced anxiety of *Tdrd3*-null mice, it is possible that it could also be a simple consequence of increased overall physical activity, as measured by total distance traveled in the open field (Fig. S3d-e).

To corroborate an anxiolytic effect and rule out hyperlocomotion as a confound, we conducted the marble burying test. In this test, anxiolytic drugs produce behavioral changes associated with reduced physical activity^50^, whereas these drugs increase activity in other test environments^51^. Accordingly, Tdrd3-null mice buried fewer marbles (Fig. 1f, main effect of genotype *F*_1,65_=10.9, *p* = 0.002). This result confirms an anxiolytic effect of knockout, rather than simple hyperlocomotion. In the elevated zero maze, *Tdrd3*-null mice showed no clear trend for increased time spent in the open quadrants (Fig. S3f). Collectively, these results indicate reduced anxiety in *Tdrd3*-null mice, a phenotype that is directly opposite to *Top3b*-null mice, which show increased anxiety in a nearly identical battery of tests.

### Tdrd3- null mice show impairments in olfactory function but no deficit in social behaviors

The buried food test requires mice to locate food using only odor cues^52^. Tdrd3-null mice were delayed in finding food (Fig. 1i, main effect of genotype *F*_1,28_=9.4, *p* = 0.005), suggesting an impairment in olfactory sensitivity. To rule out motivational effects on food retrieval latency, we performed a control experiment with food placed on the surface of the bedding, thus allowing use of visual cues in locating food. In this experiment, *Tdrd3*-null mice were not impaired in food retrieval latency (main effect of genotype *F*_1,28_=2.124, *p* = 0.15), however there was a trend in males to be delayed in feeding even when the food was visible (Fig. 1g; main effect of genotype: F_1,28_=2.124, p = 0.15; main effect of sex: F_1,28_=2.824, p = 0.10). This is consistent with *Top3b*-null mice, which performed normally in this task^11^. Conversely, *Tdrd3*-null mice were normal in two tests of social behavior in which *Top3b*-null mice showed abnormalities (Fig. S3h,i). Moreover, *Tdrd3*-null mice were normal in acoustic startle and prepulse inhibition, identical to behaviors of *Top3b*-null (Fig. S3j-k). The findings that *Top3b*-null and *Tdrd3*-null mice have shared and unique behavior phenotypes.

### Tdrd3- null mice have impaired hippocampal synaptic plasticity

The findings that *Tdrd3*-null and *Top3b*-null mice share the same phenotype of impaired hippocampus-dependent cognition imply that the former may also resemble the latter in defective hippocampal synaptic plasticity^11^, which is known to disrupt cognition^53^. To investigate this suggestion, we performed electrophysiological recordings of CA1 neurons in hippocampal slices of *Tdrd3*-null mice using the same assays previously applied to *Top3b*-null mice. These include assays of long-term potentiation (LTP) and long-term depression (LTD), both of which are broadly used to study activity-dependent long-lasting changes in synaptic plasticity^54^. In the LTP assay, *Tdrd3*-null mice exhibited a significantly decreased EPSP slope (about 30%) in response to high-frequency stimulation compared to WT mice (Fig. 1j). For LTD, *Tdrd3*-null mice also displayed reduced depression in response to a low-frequency stimulus (about 50% of that of WT mice) (Fig. 1k). The observed reduction of LTP and LTD in *Tdrd3*-null cells is weaker than that in *Top3b*-null mice, which showed nearly 80% and 100% reduction in each assay, respectively^11^. However, the findings that *Tdrd3*-null and *Top3b*-null mice both have decreased synaptic plasticity may account for the hippocampus-dependent cognitive dysfunction observed in their behavior assays. That Tdrd3-null mice have weaker defects in LTP and LTD than *Top3b*-null mice is consistent with the Morris water maze: the cognitive defect of *Tdrd3*-null mice is less than that of *Top3b*-null mice, which showed significant impairments as early as the training phase.

We also performed a short-term synaptic transmission assay, paired-pulse facilitation (PPF)^55^, and observed no significant difference between *Tdrd3*-null and WT mice (Fig. S3l). The results are similar to those with *Top3b*-null mice^11^, suggesting that depletion of either protein does not affect the transmitter release triggered by presynaptic Ca^2+^ concentration^55, 56^.

### Tdrd3 -null mice exhibit defective adult neurogenesis

**Figure 2:**
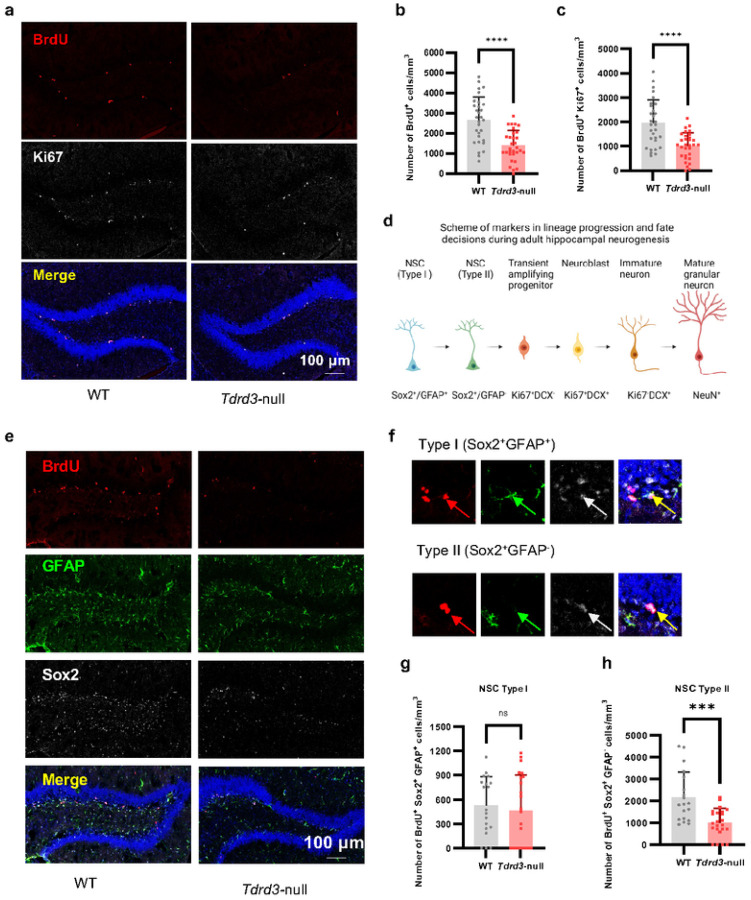
Adult neurogenesis is decreased in subgranular zone of mouse hippocampus in *Tdrd3*-null mice, especially for the type II subtype neural stem cells. **a-c** Images (a) and their Quantifications (b-c) to show that proliferation of aNSCs in *Tdrd3*-null mice were reduced significantly compared to WT mice. This is indicated by decreased density of BrdU^+^ cells (b) and BrdU^+^Ki67^+^ cells (c) in BrdU pulsed-labeling experiments. Color codes in (a): Red, BrdU; White, Ki67; Blue, DAPI. Mouse numbers: WT=4, *Tdrd3*-null=4, with 7–8 slices/mouse. **d** A scheme of lineage-specific markers during hippocampal neural stem cell development. The cells at different developmental stages are shown at the top, whereas their markers are shown at the bottom. **e-h** Images (e-f) and their Quantifications (g-h) to show that the type II but not type I aNSCs in SGZ are compromised in *Tdrd3*-null mice. This is revealed by decreased BrdU^+^Sox2^+^GFAP^−^ cell density (e, f, h) but unaltered BrdU^+^Sox2^+^GFAP^+^ cell density (g) (Red, BrdU; Green, GFAP; White, Sox2; Blue, DAPI). Number of mice used: WT=4, *Tdrd3*-null =4, with 4–6 slices/mouse. Data are presented as mean values + SD. Two-tail Student’s t test was performed for (b), (c), (g), (h). *p*-values < 0.001, and 0.0001 are marked as: ***, and ****; *p*-value > 0.5 is marked as ns.

The results that *Tdrd3*-null mice mimic *Top3b*-null mice in defective LTP, LTD and cognition^11^ promoted us to investigate whether the former also resemble the latter in defective hippocampal adult neurogenesis, which is known to be important for mood and spatial learning and memory^11, 57, 58^. We investigated whether proliferation of adult neural stem cells (aNSCs) in the subgranular zone (SGZ) of hippocampus of Tdrd3-null mice is reduced, using the same bromodeoxyuridine (BrdU)-labeling assay^36^ that had been utilized for *Top3b*-null mice. We found that the density of BrdU-labeled aNSCs is significantly lower (about 50%) in *Tdrd3*-null vs. WT mice (Fig. 2a–b), identical to the phenotype of *Top3b*-null mice^11^, indicating that the entire Top3b-Tdrd3 complex is required for adult hippocampal neurogenesis. In support of this inference, we performed double-staining with BrdU and another cell proliferation marker, Ki67, and obtained nearly identical results—the density of double-positive cells was decreased about 50% in *Tdrd3*-null mice (Fig. 2a, c).

To determine which type of aNSC is defective in proliferation (Fig. 2d), we discriminated aNSCs by triple staining cells with BrdU and two unique markers for type I and II aNSCs, GFAP and Sox2^59^, respectively. We found that the type II aNSCs (marked by Sox2^+^/GFAP^−^) exhibited about 50% reduction, whereas type I cells (marked by Sox2^+^/GFAP^+^) remained unchanged (Fig. 2e–h), indicating that *Tdrd3* inactivation Specifically disrupts proliferation of type II aNSCs. We failed to detect any apoptotic cells by TUNEL staining in either *Tdrd3*-null or WT mice (Fig. S4), thus decreasing the possibility of increased apoptosis accounting for the reduction in number of aNSCs.

### Tdrd3 -null mice have abnormal newborn neurons and dendritic spines

**Figure 3:**
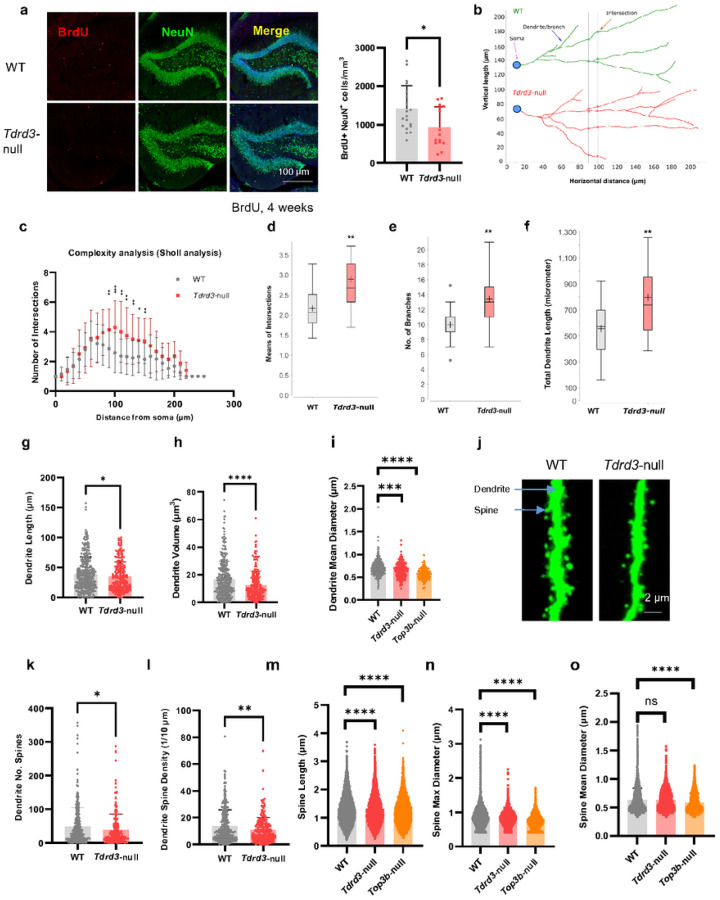
Neuronal complexity and spine morphology are altered in newborn hippocampal neurons of *Tdrd3*-null mice while only spines are altered in *Top3b*-null mice. **a** Images (left) and their Quantifications show that the newborn neuron density is decreased in *Tdrd3*-null mice. This is demonstrated by lower BrdU^+^NeuN^+^ cell density in *Tdrd3*-null mice than that in WT mice, 4 weeks after BrdU labeling (Red, BrdU; Green, NeuN; Blue, DAPI). Mouse numbers: WT=4, *Tdrd3*-null =4, with 3–4 slices/mouse. **b-c** Sholl complexity analysis indicates that the newborn neurons are more complex in *Tdrd3*-null than WT mice. This is illustrated by larger numbers of intersections at distances from soma from 90 to 150 μm in *Tdrd3*-null than WT mice. **d-f** Graphs from sholl analysis show that the mean intersection numbers (d), branch numbers (e) and total dendrite lengths (f) are all increased in neurons of *Tdrd3*-null mice vs. WT control mice. **g-h** The dendrites are smaller and shorter in *Tdrd3*-null mice, which are indicated by reduced dendrite lengths (g) and dendrite volumes (h) in *Tdrd3*-null mice. **i** The dendrites are thinner in both *Tdrd3*-null and *Top3b*-null mice, which are demonstrated by decreased dendrite mean diameters. **j** Representative images show that the spine density and sizes are smaller in *Tdrd3*-null mice than WT mice. **k-l** Graphs show that spine numbers in each dendrite (k) and dendrite spine density (l) are decreased in *Tdrd3*-null mice. **m-n** Graphs show that both spine lengths and spine max diameters are decreased in *Tdrd3*-null and *Top3b*-null mice. **o** A graph shows that the spine mean diameters are significantly reduced in *Top3b*-null mice, but remain unchanged in *Tdrd3*-null mice. Data are presented as mean values + SD (a, g-i, k-o), mean values ± SD (c-f). Two-tail Student’s t test was performed for all comparisons. *p*-values < 0.05, 0.01, 0.001, and 0.0001 are marked as: *, **, ***, and ****; *p*-value > 0.5 is marked as ns.

Our findings that *Tdrd3*-null mice have defective proliferation of aNSCs raised the possibility that they have abnormal newborn neurons, which are known to experience a series of developmental changes after birth^60, 61^. To assess this possibility, we injected mice with BrdU and euthanized them after a 4 week interval to allow the dividing progenitor cells to develop into mature new neurons. We then examined the newborn mature neurons in SGZ of hippocampus in *Tdrd3*-null mice by double-staining with BrdU and a mature neuron marker, NeuN. We found that the density of double-positive cells (BrdU^+^NeuN^+^) is significantly reduced (about 40%) in *Tdrd3*-null mice (Fig. 3a), indicating that the number of newborn mature neurons in null mice is decreased, consistent with the data that the null mice have defective proliferation of aNSC.

We subsequently investigated the morphology of newborn neurons in the dentate gyrus (DG) of hippocampus by GFP-retroviral labeling^11, 60^. We then performed neurite tracing and complexity analysis on the obtained images. We observed several abnormal features in *Tdrd3*-null mice vs. WT controls. First, the null mice displayed a larger number of intersections in the middle (90–150 mm away from soma) (Fig. 3b–c, S5a) and total lengths of neurites (Fig. 3d), as well as an increased number of branches (Fig. 3e), indicating increased neuron complexity. Second, the average lengths of total neurites were significantly increased (Fig. 3f), whereas the average lengths of single neurite were decreased (Fig. 3g). This difference could be explained by the increased number of branches in *Tdrd3*-null mice (Fig. 3d), so that their neurites are shorter but more numerous. Third, the volumes, lengths, and thickness of dendrites were all decreased in null mice (Fig. 3h–i). Collectively, these results demonstrate that *Tdrd3*-null mice have abnormal numbers and morphology in newborn neurons, in accord with the findings that their adult neurogenesis is defective.

Dendritic spines from adult newborn neurons are crucial for the synaptic plasticity underlying learning and memory^62, 63^, and spine abnormalities have been observed in patients and animal models of neurological diseases^64^, including *Top3b*-null mice^11^. We found that spines of *Tdrd3*-null mice resemble *Top3b*-null in several abnormal features, including reduced spine numbers in each dendrite^11^ (Fig. 3k); lower spine density^11^ (Fig. 3l); shorter lengths (Fig. 3m); and smaller maximum diameter (Fig. 3n). However, *Tdrd3*-null mice exhibited no significant reduction in the mean diameter of spines, unlike *Top3b*-null mice^11^ (Fig. 3o). The observed abnormality in spines of hippocampal neurons of both *Tdrd3* and *Top3b*-null mice could contribute to their defective synaptic plasticity and cognitive function.

We previously reported that the newborn neurons of *Top3b*-null mice have decreased complexity^11^. However, that finding was not based on the more sensitive neurite tracing and quantitative analysis of images used in this study. We reanalyzed our previous data^11^ using the current method, and found that *Top3b*-null mice exhibited no significant difference in complexity vs. WT based on the normal number of intersections (Fig. S5b-c), branches (Fig. S5d), and dendrite length (Fig. S5e), This conclusion differs from that for *Tdrd3*-null mice, which show increased complexity. Nevertheless, the newborn neurons of *Tdrd3*-null mice share several features with *Top3b*-null mice including reduced dendrite mean diameter (Fig. 3i) and smaller dendrite length and volume^11^ (Fig. 3g, 3h). The data reinforce the notion that *Tdrd3* and *Top3b* -null mice have both shared and unique phenotypes.

### Tdrd3- null mice exhibit reduced axon myelination

**Figure 4:**
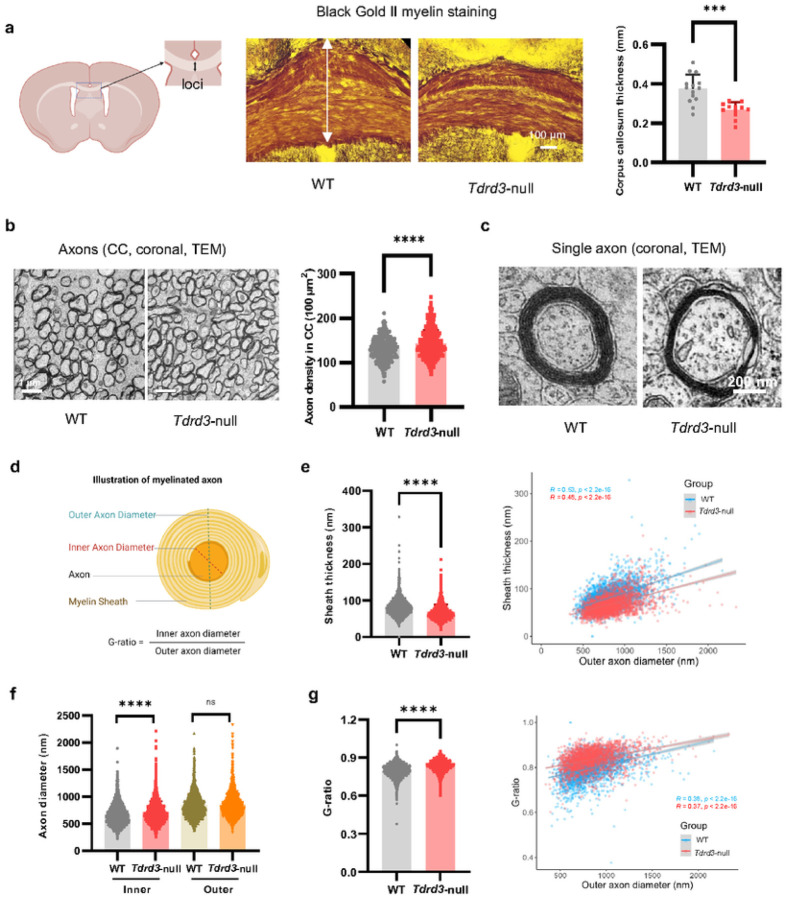
*Tdrd3*-nullmice show reduced corpus callosum thickness and increased axon density in the brain. **a** Images (middle) and their Quantifications (right) from Black Gold II staining show that the thickness of corpus callosum (marked by the arrow) is significantly reduced in *Tdrd3*-null mice. A picture showing the locus of corpus callosum is shown on the left. **b** Electron micrographs (left) and their Quantifications show that axon density in corpus callosum (CC) is increased in *Tdrd3*-nullmice. **c-d** Enlarged electron micrographs (c) show that single axons from *Tdrd3*-*null*mice have reduced thickness in myelin sheath. Illustration of myelinated axons in CC was shown in (d). (**e-g**) Quantification of electron micrographs show differences between Tdrd3-null and WT mice in sheath thickness (e), inner and outer diameters of axons (f), and G-ratios. e The left graph shows that the average sheath thickness is decreased in *Tdrd3*-null mice. The right scatter plot shows that the sheath thickness has modest positive correlation with the outer axon diameters. The correlation coefficient (R) and p-values are listed in the graphs. The datapoints and the trendline from Tdrd3 null mice are largely lower than those of WT mice, indicating reduced sheath thickness in the former animals. **f** A graph shows that the Inner axon diameter is longer, whereas the outer axon diameter is not significantly different, in *Tdrd3*-null mice when compared to those of WT mice. **g** The left graph shows that the G-ratios are significantly higher in *Tdrd3*-null mice than WT. The right scatter plot shows that the G-ratio has weak positive correlation with outer axon diameters. The datapoints and trendline from *Tdrd3*-null mice are largely higher than those of WT mice, indicating that axons from the former mice have larger G-ratios (meaning reduced sheath thickness). Data are presented as mean values + SD. Two-tail Student’s t test was performed for all comparisons. Linear model t was used for fitted curves in e (right) and g (right). *p*-values < 0.001, and 0.0001 are marked as: ***, and ****; *p*-value > 0.5 is marked as ns.

The reduced anxiety and impaired cognitive behaviors observed in *Tdrd3*-null mice resemble effects seen in rodents following demyelination of the corpus callosum (CC)^65, 66^. In addition, reduced myelination has been reported in patients and animal models of autism and schizophrenia^65, 67, 68^, both of which have been associated with *Top3b* mutations^2^. We therefore examined myelination in *Tdrd3*-null mice by Black Gold II myelin staining, observing significantly reduced CC thickness (Fig. 4a). This reduction could be due to decreased myelination, and/or increased density of axons. To distinguish between these alternatives, we further examined CC by transmission electron microscopy (TEM) and observed an increase of axon density in the null mice (Fig. 4b). Notably, the thickness of the myelin sheath was significantly decreased in *Tdrd3*-null mice (Fig. 4c–d; 4e, left), and this decrease was more obvious when the thickness was plotted vs. the outer axon diameter (Fig. 4e, right; more data points (red) and the trendline from *Tdrd3*-null, below those of WT (blue)), indicating reduced myelination. Consistent with this conclusion, the inner axon diameter of myelinated axons was significantly increased, whereas the outer axon diameter remained unchanged (Fig. 4f). This was also reflected by a significant increase of the G-ratio (Fig. 4g, left), which is calculated as the ratio between the inner and outer axon diameters^69^, and has been extensively utilized as a structural and functional index of optimal axonal myelination^70^. The increase of G-ratios was also more evident when it was plotted vs. outer axon diameter (more datapoints and trend line from *Tdrd3*-null cells are above those of WT (Fig. 4g, right). Together, these data suggest that *Tdrd3*-null mice have reduced myelination and increased axon density in CC, both of which may contribute to the reduced anxiety and impaired cognitive behaviors.

### Tdrd3- null mice display impaired neuronal activity- dependent transcription

We next explored whether the *Tdrd3*-null mice exhibit defects in neuronal activity-dependent transcription (NADT) of immediate early genes (IEGs) in response to fear conditioning stress as do *Top3b*-null mice^11^. Here we analyzed *Tdrd3*-null mice treated with the same stimulus and observed significantly reduced induction of several IEG mRNAs in amygdala and hippocampus (Fig. S6), two brain regions critical for fear memory^71, 72^. More IEG mRNAs showed significant reduction in amygdala than hippocampus (7 vs. 3 among 9 genes tested) by RT-qPCR, suggesting that Top3b-Tdrd3 may be more important in the former than the latter regions for NADT. In contrast, no tested IEGs were differentially altered in the cortex. The findings that both *Tdrd3*-null and *Top3b*-null mice exhibit reduced induction of IEGs in response to fear conditioning suggest that the entire Top3b-Tdrd3 complex is needed for NADT.

### Tdrd3 -null mice exhibit abnormal post-transcriptional regulation

**Figure 5:**
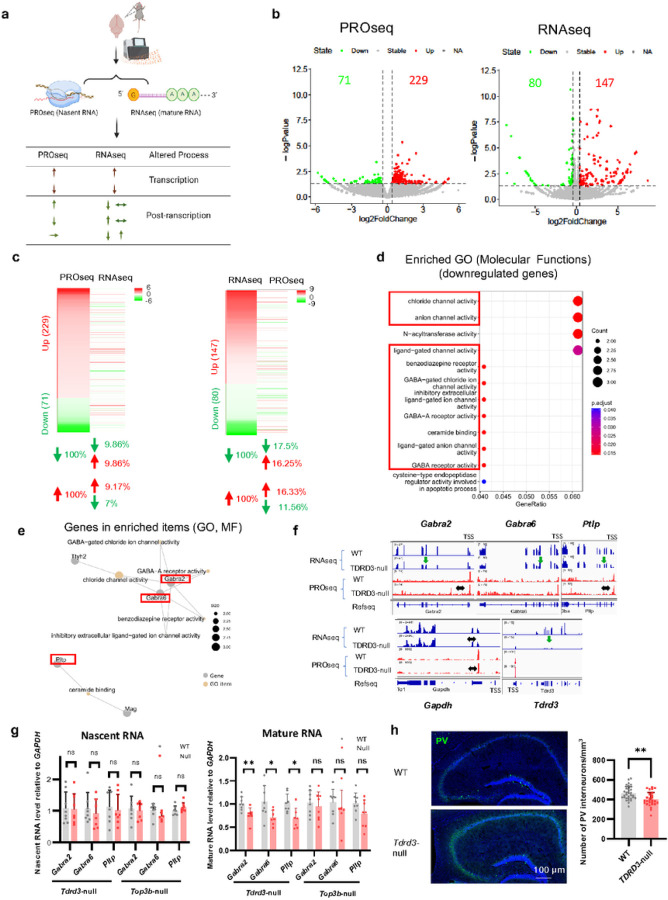
Several mRNAs associated with GABAergic interneurons are downregulated by post-transcriptional mechanisms in *Tdrd3*-null mice. **a** A schematic diagram of experimental design depicts that nascent and mature mRNAs are pro led by PROseq and RNAseq, respectively. The former method detects mRNA altered at the transcriptional step, whereas the latter measures mRNA changes caused by both transcriptional and post-transcriptional mechanisms. The up or down arrows indicate differentially expressed genes (DEGs) that are increased or decreased in null mice, respectively, whereas the horizonal arrows mark mRNAs that remain unchanged. The mRNAs that do not show the same direction of alteration in both methods should be regulated by post-transcriptional mechanism (such as turnover). **b** Volcano plots show differentially expressed genes (DEGs) identified by PROseq (left) and RNAseq (right) in *Tdrd3 null* mouse brains (green, downregulated; red, upregulated, log2FoldChange > 1.3 and *p*-value < 0.05). **c** HEATmap analysis to compare DEGs obtained by PROseq vs. those by RNAseq. The left panel illustrates how the up or down-regulated DEGs from PROseq are altered in RNAseq. The right panel shows the reciprocal comparison: how the up and down-regulated DEGs from RNAseq are altered in PROseq. The data lines marked in the same color indicate DEGs that are altered in the same direction by both assays. The table below the HEATmaps indicate that the percentages of DEGs that are altered in the same or opposite directions, as marked by the directions of arrows. **d** Gene ontology analysis of downregulated DEGs from RNAseq using molecular function category indicates that the expression of genes associated with chloride channels in GABAergic interneurons (marked by red boxes) is disturbed in *Tdrd3*-nullmice. **e** Relationship between several enriched GO terms and their associated genes from (d). **f** Graphs from USCS genome browser show that several representative genes associated with chloride channels exhibit reduced signals by RNAseq but unchanged signals by PROseq (*Gabra2*, *Gabra6*, *Pltp*) in *Tdrd3*-null mice. The down arrows mark the RNAseq signals thar are reduced in the null mice, whereas the horizontal arrows mark the PROseq signals that are unchanged. *Gapdh* and *Tdrd3* genes are included as negative and positive controls, respectively. **g** RT-qPCR results of nascent RNA (left) and mature RNA (right) expression of *Gabra2*, *Gabra6* and *Pltp* in brains of *Tdrd3*-null mice and *Top3b*-null mice. **h** Immunofluorescent images (left) and their Quantification (right) show that the density of parvalbumin positive cells is significantly reduced in *Tdrd3*-nullmice. Data are presented as mean values + SD. Two-tailed Student’s t test was performed for the comparisons. *p*-values < 0.05, 0.01 are marked as: *, **; *p*-value > 0.5 is marked as ns.

Top3b-Tdrd3 has been reported to participate directly in both transcriptional^11, 16, 17^ and post-transcriptional regulation^21^. The evidence for the latter includes defective mRNA translation and turnover^21^ in HCT116 null cells for either protein. To determine how *Tdrd3* regulates brain function and whether *Tdrd3*-null mouse brains exhibit abnormal mRNA turnover, we employed a strategy used by previous studies^16, 73^, comparing the levels of nascent RNA determined by PROseq *versus* those of mature RNA by RNAseq. The former is a readout of genome-wide transcription, whereas the latter is the result of both transcription and mRNA turnover; and differences between the two should be due to mRNA turnover (Fig. 5a).

Our PROseq and RNAseq analyses of nascent and mature mRNAs from whole brains of *Tdrd3*-null and WT control mice identified 300 and 227 differentially-expressed genes (DEGs) (p < 0.05, fold change 1.3), respectively (Fig. 5b; Table S1), suggesting that Tdrd3 regulates only a small fraction of genes at transcription and/or post-transcriptional steps^21^. In both sequencing analyses, the DEGs that show increase of their levels in null mice (229 and 147, respectively) are about 2–3 fold greater than those that show decrease (71 and 80, respectively), suggesting that Tdrd3 can either positively or negatively regulate expression of a small fraction of genes. These data are largely consistent with our previous findings in human KO cell lines of either *Tdrd3* or *Top3b*, as well as those from *Top3b*-null mice^11, 16, 21^.

Comparison of DEGs between PROseq and RNAseq by heatmaps revealed substantial differences (Fig. 5c; Table S2,S3). For example, for DEGs that show either an increase or decrease by PROseq in *Tdrd3*-null mice, the percentages of them showing the same direction of alteration by RNAseq are fewer than 10%, whereas the majority remain unchanged (~ 80%), with a smaller fraction (< 10%) showing oppositive direction of alteration (Fig. 5c, left; Table S2, S3). Similarly, For DEGs showing an increase or decrease by RNAseq, the percentages of them showing the same direction of alteration by PROseq are fewer than 20%, whereas the majority (> 65%) remained unchanged, and a smaller fraction exhibited the opposite direction of alteration (< 17%) (Fig. 5c, right; Table S2, S3). The findings that the majority of DEGs altered by one assay are not altered in the same direction by the other suggest that *Tdrd3* inactivation in mouse brains has a major effect on mRNA turnover, with only a small effect on transcription.

We also analyzed the RNAseq data from *Top3b*-null mouse brains^11^, and identified 156 increased and 69 decreased DEGs vs. WT control mice (Fig. S7a,b). Unexpectedly, the percentage of overlapped DEGs between *Top3b*-null and *Tdrd3*-null mice are low (< 13% of total DEGs), regardless whether they are increased or decreased (Fig. S7c-d). These data differ from findings from HCT116 cells in which the percentages of overlapped DEGs were much higher (50% and 30% for increased and decreased DEGs, respectively^21^). This difference between adult brains of the two null mice may be caused by different functions of each protein in earlier development. More work is needed to determine whether newborn or fetal brains from these mice are as different as are the adult brains.

### Tdrd3 -inactivation alters the turnover rates of mRNAs encoding each of several GABA receptors

To determine which mRNAs altered in *Tdrd3*-null mice may account for the observed behavioral and neurological abnormality, we performed Gene Ontology (GO) analysis of DEGs from RNAseq. Molecular functional analysis of DEGs with reduced expression in *Tdrd3*-null mice identified several enriched GO terms that are associated with GABA-gated chloride channels or ceramide binding (Fig. 5d). We examined DEGs involved in these GO terms and selected three genes: *Gabra2, Gabra6*, and *Pltp* (Fig. 5e), for further analysis. UCSC genome browser analysis revealed that RNAseq signals for these genes were reduced (about 47%, 35.4% and 29.6%) in *Tdrd3*-null mouse brains (based on average TPM), whereas their PROseq signals remain either unchanged (< 1.2 fold difference, increased 16.8%, 0) or increased (*Gabra6*, 2.7 fold) (Fig. 5f). As negative controls, the signals of both RNAseq and PROseq for Gapdh were unchanged. As a positive control, the RNAseq signal for Tdrd3 was significantly reduced (reduced 10.4 fold) in *Tdrd3*-null mice, consistent with RT-qPCR data (Fig. S2d). RT-qPCR analysis confirmed that mature mRNA levels for *Gabra2*, *Gabra6*, and *Pltp* were reduced by about 30% (p < 0.05) (Fig. 5g, right), whereas their precursor mRNA levels remained unchanged (Fig. 5g, left), in *Tdrd3*-null mice. Apparently, mature mRNA levels of these gene are reduced in *Tdrd3*-null mice; and this reduction is not due to reduced transcription, but rather accelerated mRNA turnover. Our data that *Gabra2* and *Gabra6* are regulated post-transcriptionally by Tdrd3 are reminiscent of earlier findings that GABA receptor mRNAs are subject to post-transcriptional regulation by multiple RBPs, including FMRP^74^. However, in *Top3b*-null mice, RT-qPCR result indicate neither of their mature and precursor RNA are not significantly altered (Fig. 5g).

Gabra2 and Gabra6 are distinct subunits of the ligand-gated chloride channel receptor for the major inhibitory neurotransmitter GABA. These subunits are vital for the formation of inhibitory GABAergic synapses^75^. The reduced expression of *Gabra2* and *Gabra6* led us to investigate whether parvalbumin (PV)-expressing GABAergic interneurons are impaired in *Tdrd3*-null mice. Immunostaining showed a marked decrease in the density of PV-positive interneurons (p < 0.05) in the hippocampus of *Tdrd3*-null mice (Fig. 5h). These data are consistent with our RNAseq data showing reduced expression of GABAergic-associated genes (*Gabra2* and *Gabra6*) in *Tdrd3*-null mice.

### Tdrd3 -null mice exhibit reduced expression of several genes downstream of GABA receptors

**Figure 6:**
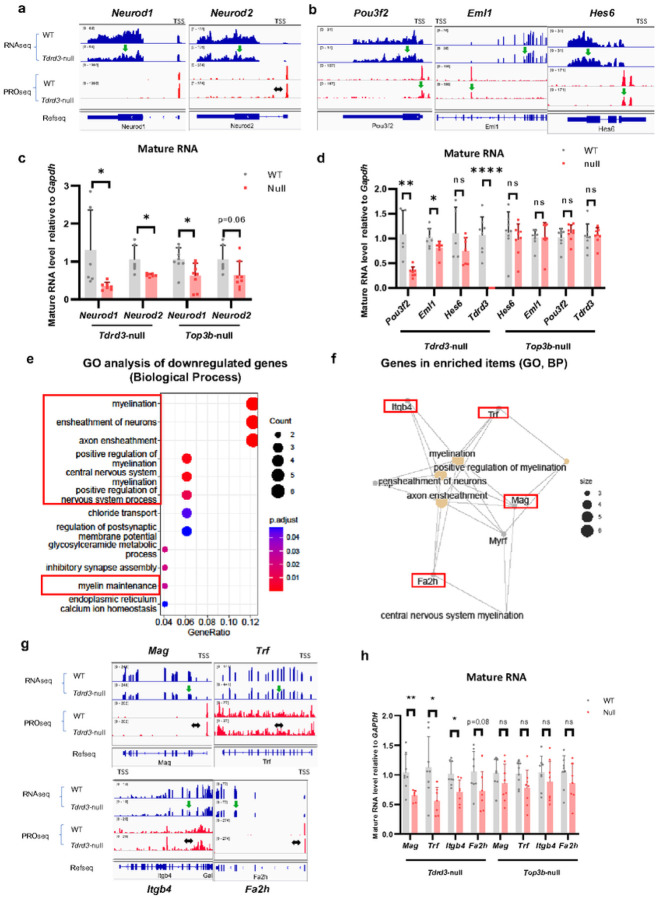
Several *Gabra2* downstream genes and myelination associated genes are downregulated by post-transcriptional mechanism in *Tdrd3*-nullmice. **a** Bedgraphs from UCSC genome browser analysis show that two genes downstream of Gabra2, *Neurod1* and *Neurod2*, exhibit reduced RNAseq signals but unchanged PROseq signals in *Tdrd3*-null mice. **b** Bedgraphs from UCSC genome browser analysis show that three genes that are known to be bound and enhanced expression by *Neurod1* exhibit reduced signals of both RNAseq and PROseq in *Tdrd3*-nullmice. The down arrows mark reduced signals, whereas horizontal arrows mark genes that are unchanged in the null mice. **c** RT-qPCR results show that mature RNA levels of *Neurod1* and *Neurod2* are significantly decreased in *Tdrd3*-null mice (p<0.05), and both genes also show a strong trend of reduction in *Top3b*-null mice (for *Neurod2*, p=0.06; which does not reach statistical significance). **d** RT-qPCR analyses show that mature RNA levels of *Eml1* and *Pou3f2*, but not *Hes6*, are significantly reduced in *Tdrd3*-nullmice. **e**. Gene ontology enrichment analysis of downregulated genes in *Tdrd3*-null mice using Biological Process category shows that several top enriched GO terms are related to myelination (marked in red boxes). **f** Relationship between enriched GO items and their associated genes reveals that these terms are closely associated with each other and share several common genes (marked as red rectangles). **g** Bedgraphs from USSC genome browser analysis of RNAseq and PROseq show that mature but not nascent RNA levels for several myelination-associated genes are reduced in *Tdrd3*-null mice (*Mag*, *Trf*, *Itgb4* and *Fa2h*). The down arrows mark reduced signals, whereas horizontal arrows mark genes that are unchanged in the null mice. **h** RT-qPCR results show that mature RNA levels of several myelination associated genes are decreased in Tdrd3-null, but not *Top3b*-null mice. Data are presented as mean values + SD. Two-tailed Student’s t test was performed for the comparisons. *p*-values < 0.05, 0.01, 0.001, and 0.0001 are marked as: *, **, ***, and ****; *p*-value > 0.5 is marked as ns.

The findings that mRNA levels of *Gabra2* and *Gabra6* are reduced in *Tdrd3*-null mice raised the possibility that other genes acting in the GABAergic pathway might show similarly reduced expression. We investigated this hypothesis and found that two genes acting in this pathway, *Neurod1* and *Neurod2*^76,77^, also display reduced mRNA levels by RNAseq(Fig. 6a) and RT-qPCR (Fig. 6c) in *Tdrd3*-null mouse brains. Thus, the signaling cascade between GABA receptors and Neurod molecules could be impaired. Analysis of PROseq and RT-qPCR data showed that nascent RNA levels of *Neurod1* and *Neurod2* are not decreased in *Tdrd3*-null mice (Fig. 6a, S8a), so that their reduced mRNA levels are likely due to accelerated turnover. This is consistent with previous data that *Neurod1* mRNA is translationally regulated by FMRP^78^, a frequent partner of the Top3b-Tdrd3 complex.

Mutations in *Neurod2* have been associated with autism^79, 80^, similar to mutations in *Top3b*. This prompted us to examine the expression of *Neurod1* and *Neurod2* in *Top3b*-null mouse brains. We observed significant reduction of *Neurod1* mature mRNA levels by RT-qPCR as in *Tdrd3*-null mice, and a strong decreased trend in *Neurod2* mRNA levels (Fig. 6c) (p = 0.06). Interestingly, we did observe statistically significant reduction of *Neurod1* nascent RNA (Fig. S8a), consistent impairment of transcription of this gene in *Top3b*-null mice.

Neurod1 and Neurod2 are pioneer transcriptional factors that establish transcriptional and epigenetic pro les in the neuronal lineage^81–83^. Hundreds of genes bound and upregulated by ectopically expressed Neurod1 in mouse ES cells have been identified^81^. We found that only a small percentage (< 0.5%) of these Neurod1-bound genes exhibited reduced PROseq or RNAseq signals in *Tdrd3*-null mice, whereas the majority (99%) remained unchanged (Fig. S8b). UCSC genome browser analysis revealed reduced PROseq signals for three representative Neurod1-bound genes (*Hes6*, *Eml1* and *Pou3f2*) (Fig. 6b). RT-qPCR confirmed statistically significant reduction of mature mRNA levels for two genes (*Eml1* and *Pou3f2*) in *Tdrd3*-null (Fig. 6d) but not *Top3b*-null mice. We did not observe the significant reduction in nascent RNA of these three genes in both *Tdrd3* and *Top3b* deficient mice (Fig. S8c). Because the nascent and mature mRNA levels of these three genes exhibit significant reduction in one but not the other sequencing assay, the data imply that these genes are also regulated by turnover.

### Tdrd3 -null mouse brains display reduced expression of several myelination genes.

To investigate how Tdrd3 inactivation leads to demyelination of axons in CC, we performed GO analysis of our RNAseq data using Biological Process Category and observed “myelination” in several top 10 enriched GO terms (Fig. 6e). Examination of these terms revealed several myelination-associated genes (*Mag*, *Trf*, *Itgb4* and *Fa2h*)(Fig. 6f) that showed reduced levels of RNAseq, but unchanged levels of PROseq(Fig. 6g), indicating that the reduced expression of these genes in *Tdrd3*-null mice is due to accelerated turnover, rather than decreased transcription. RT-qPCR analysis confirmed that three of the 4 myelination genes exhibited significantly reduced levels of mature mRNA (Fig. 6h); and all 4 genes display normal levels of nascent RNAs (Fig. S8e). As a comparison, the same 4 myelination genes did not show significant changes in their mature or pre-mRNA levels by RT-qPCR in Top3b-null mouse brains (Fig. 6h, S8e), indicating that reduced expression of myelination genes is limited to *Tdrd3*-null mice. Because inactivation of some of these myelination-associated genes (*Itgb4*, *Fa2h* and *Trf*) can cause demyelination^84–86^, their reduced expression can explain the demyelination phenotype observed in *Tdrd3*-null mice.

## Discussion

### Tdrd3 -null mice display some neurological and behavior problems similar to Top3b-null mice.

A major objective of this study is to establish a mouse model to examine a hypothesis that Tdrd3 may resemble its two partners (Top3b and FMRP) in playing important roles in psychiatric and cognitive disorders. This hypothesis is supported by human GWAS data that several SNVs of TDRD3 genomic locus are associated with these disorders. Our findings that *Tdrd3*-*null* mice resemble *Top3b*-*null* mice in exhibiting abnormality in cognitive and anxiety behaviors, synaptic plasticity, adult neurogenesis, neuron morphology, and demyelination indicate that Tdrd3 could play similar roles in psychiatric and cognitive disorders as do its interacting partners Top3b and FMRP. Moreover, our data suggest that the entire Top3b-TDRD3 complex and its partner FMRP are required for normal brain development and function.

Tdrd3 is a regulatory subunit of the Top3b-Tdrd3 topoisomerase complex, recruiting Top3b to its target genes through Tudor and other protein-interaction domains, enhancing the topoisomerase activity of Top3b, and stabilizing Top3b protein. Another objective of the current study is to address a question whether Tdrd3 acts only as a cofactor of Top3b or has its own functions. We did observe behavioral and neurological defects in *Tdrd3*-null mice that overlap with those of *Top3b*-null mice (summarized in Fig. S9a). The defects observed in both knockout models include spatial memory, conditioned fear (enhanced), synaptic plasticity (LTP and LTD), proliferation of adult neural stem cells, dendrite size, spine density and size, and neuronal activity dependent transcription. The phenotypes overlapping between *Tdrd3*-null and *Top3b*-null mice are consistent with our earlier studies of their KO cell lines^21^, which also revealed overlap in their differentially-expressed genes. The overlapping phenotypes in both mouse and cell models argue that a main function of Tdrd3 is to facilitate Top3b action.

### Tdrd3 -null mice have several phenotypes distinct from those of Top3b-null mice

Though *Tdrd3*-null and *Top3b*-null mice share many effects (Fig. S9a), differences between the two mice are also evident. For example, *Tdrd3*-null mice exhibit reduced anxiety in several behavior assays, which is opposite to *Top3b*-null mice, which show increased anxiety. In addition, *Tdrd3*-null mice showed normal social interactions, unlike the deficits observed in *Top3b*-null mice. Conversely, mice null for *Tdrd3* but not for *Top3b* have impaired olfactory function. Moreover, *Tdrd3*-null mice show increased complexity of newborn neurons, whereas *Top3b*-null mice do not. These differences in behavior and neuron morphology likely result from the differences in gene expression pro les in two knockout strains, which show very limited overlap of upregulated or downregulated DEGs under steady state conditions (Fig. S7c,d).

The differences observed between gene expression pro les are supported by RT-qPCR analysis showing that multiple genes important for neural development and function are altered in *Tdrd3*-null but not *Top3b*-null mice (*Graba2*, *Gabra6*, etc; Fig. 5–6). These data are consistent with RNAseq and Ribo-seq analysis of their KO cell lines, which revealed differences in gene expression pro les^16, 21^. It is possible that although Top3b and Tdrd3 are component forms of a complex, each subunit may have some functions independent of its partner. Suggestively, Tdrd3 contains multiple protein-interacting domains^1^ and can directly interact with a variety of DNA and RNA processing molecules (RNA Pol II, histone tails, FMRP, and exon junction complex). Conversely, Top3b contains an mRNA binding activity that depends on a conserved RNA-binding domain (RGG-box), but not the catalytic residue (Y336); which could function independently of Tdrd3. Taken together, these data indicate that Tdrd3 and Top3b have both shared and unique functions within their complex.

### Tdrd3 Regulates Gene Expression At Both Transcriptional And Post-transcriptional Steps

Earlier work based on *Top3b*-null mice indicates that the complex can act on DNA transcription: it can directly enhance neuronal activity-dependent transcription of neuronal early response (NER) genes^11^. Our current findings that several NER genes show reduced neuronal-activity dependent transcription in tissues from *Tdrd3*-null mouse brains further support this action (Fig. S6).

Recent work using *Top3b* and *Tdrd3*-null cell lines demonstrate that Top3b-Tdrd3 also binds to mRNAs and regulates their translation and turnover^21^. However, whether the complex can regulate gene expression in animals by post-transcriptional mechanisms has remained unclear. Here we performed gene expression pro ling of both nascent and mature mRNAs in *Tdrd3*-null mouse brains and observed that many genes important for neuronal function exhibit reduced levels in mature mRNAs but are unchanged in nascent RNA (vice versa). This indicates that turnover rates are altered in absence of Tdrd3. We noticed that some mRNAs (*Neurod1*, *Neurod2*) showed significant reduction in *Tdrd3*-null mice (p < 0.05) and also displayed a strong trend of reduction in *Top3b*-null mice (Fig. 6c), suggesting that their turnover rate may be affected by the entire Top3b-Tdrd3 complex.

### Tdrd3- null mice have defective GABA-Neurod signaling cascade

GABAergic signaling plays an indispensable role in neural development by regulating adult neurogenesis, and its disruption has been linked to autism and schizophrenia^87–89^, diseases associated with *Top3b* mutations. The finding of defective parvalbumin interneurons also suggests that GABAergic pathways were possibly compromised, consistent with decreased GABAergic inhibitory transmission in mature DG neurons^90^. Neurod transcription factors have been identified as downstream effectors of GABA cascade, as expression of *Neurod* is increased upon GABAergic excitation^76^, and decreased by GABA receptor inactivation^91^. Moreover, mutation of *Neurod2* has also been associated with autism^80^. Notably, we found that multiple mRNAs important in the GABA-Neurod signaling cascade show accelerated turnover in *Tdrd3*-null mouse brains (Figs. 5 and 6; summarized in Fig. S8d). These include mRNAs of *Gabra2*, *Gabra6*, *Neurod1* and *Neurod2* as well as some targets of Neurod1: Pou3f2, and Eml1. It has been shown that GABA Receptor mRNAs, including those of *Gabra2* and *Gabra6*; as well as *Neurod* mRNAs, are subject to post-transcriptional regulation by various RBPs, including FMRP (which directly interacts with Tdrd3). *Fmr1*-KO mice do share common phenotypes with *Tdrd3*-null and *Top3b*-null mice, including impaired adult neurogenesis, and hippocampus-dependent learning^92, 93^. Tdrd3 and Top3b may thus work with FMRP and other RBPs to regulate mRNAs important for GABAergic signaling by post-transcriptional mechanisms (Fig. S9b). Mice carrying mutations in different domains of each protein should help to elucidate their cooperative and independent actions in causing impaired adult neurogenesis, defective synaptic plasticity, impaired cognitive function, and increased risk of psychiatric disorders.

## Data Availability

All relevant next generation sequencing data are deposited at GEO database (Accession number GSE223568).
